# Evidence-Based Recommendations in Primary Tracheoesophageal Puncture for Voice Prosthesis Rehabilitation

**DOI:** 10.3390/healthcare12060652

**Published:** 2024-03-14

**Authors:** Miguel Mayo-Yáñez, Alejandro Klein-Rodríguez, Aldán López-Eiroa, Irma Cabo-Varela, Raquel Rivera-Rivera, Pablo Parente-Arias

**Affiliations:** 1 Department of Otorhinolaryngology—Head and Neck Surgery, Complexo Hospitalario Universitario de A Coruña (CHUAC), 15006 A Coruña, Spain; irma.cabo.varela@sergas.es (I.C.-V.); pablo.parente.arias@sergas.es (P.P.-A.); 2 Otorhinolaryngology—Head and Neck Surgery Research Group, Institute of Biomedical Research of A Coruña, (INIBIC), Complexo Hospitalario Universitario de A Coruña (CHUAC), Universidade da Coruña (UDC), 15006 A Coruña, Spain; alejandro.klein.rodriguez@sergas.es (A.K.-R.); aldan.lopez.eiroa@sergas.es (A.L.-E.); 3 Department of Otorhinolaryngology—Head and Neck Surgery, Hospital Público da Mariña, 27880 Lugo, Spain; 4 Department of Otorhinolaryngology—Head and Neck Surgery, Complexo Hospitalario Universitario de Ferrol (CHUF), 15405 Ferrol, Spain; 5 Atos Medical Clinical Department, Atos Medical Spain S.L., 08007 Barcelona, Spain; raquel.rivera@atosmedical.com

**Keywords:** tracheoesophageal puncture, alaryngeal, voice, prosthesis, laryngectomy, head neck, cancer, rehabilitation

## Abstract

Head and neck cancer, the seventh most common cancer worldwide, often affects the larynx, with a higher incidence in men. Total laryngectomy, a common treatment, results in the loss of phonation, and tracheoesophageal voice rehabilitation is the current rehabilitation method of choice. Despite ongoing debates regarding the timing of tracheoesophageal puncture (TEP), a crucial procedure for voice prosthesis placement, the secondary puncture continues to be the preferred choice in the majority of cases. This underscores the persistent controversy and the absence of consensus in this field. The aim of this manuscript was to define evidence-based recommendations regarding the procedure of primary TEP with voice prosthesis placement, establish the conditions and requirements for performing primary TEP, determine the indications and contraindications of primary TEP, as well as to define the complications and management of primary TEP. A total of 19 statements were formulated, with 78.95% of them having a Level of Evidence 4 and a Grade of Recommendation C. There is not sufficient evidence comparing the outcomes of primary TEP versus secondary TEP. Future studies with robust methodologies are needed to clarify the role of primary and secondary TEP in the rehabilitation of patients undergoing total laryngectomy.

## 1. Introduction

Head and neck cancer is the seventh most common cancer worldwide [1], with the larynx being the most common site in Europe, with an incidence of 4.6/100,000 [2]. This type of cancer is more prevalent in men than in women, with a ratio of 10:1, although in recent years, there has been an increase in the number of cases in women due to the rise in tobacco and alcohol consumption [3]. Generally, laryngeal cancer tends to occur more frequently between the sixth and seventh decades of life.

The therapeutic approach to laryngeal and hypopharyngeal carcinoma is multidisciplinary and involves various treatment modalities, including surgery, radiotherapy, chemotherapy, and immunotherapy, used individually or in combination, depending on the tumor stage [4]. In advanced stages of the disease, total laryngectomy may be necessary, either as the primary treatment option or as a salvage procedure after prior oncological medical treatment. This intervention involves the complete removal of the larynx and the creation of a permanent respiratory stoma, resulting in the loss of the physiological ability for phonation, which is the most significant sequel of total laryngectomy.

Since the first laryngectomies described 150 years ago [5], one of the most important goals of research in this field has been to compensate for this loss, improving patient support and comprehensive care [6,7,8,9]. Tracheoesophageal voice rehabilitation is the current method of rehabilitation of choice or gold standard therapy [10], with a 95% long-term success rate and vocal quality ranging from fair to excellent in 88% of cases [9,11]. This rehabilitation modality provides better outcomes in terms of quality of life, vocal quality, fundamental frequency, maximum phonation time, and intensity compared to esophageal voice rehabilitation [6,12,13,14]. Furthermore, studies have demonstrated good perception of vocal quality and acceptance by the patient compared to laryngeal voice rehabilitation in healthy patients [15].

The placement of a voice prosthesis involves performing a tracheoesophageal puncture (TEP), a surgical procedure that can be carried out during laryngectomy (after the removal of the larynx and before pharyngeal closure), known as primary TEP, or it can be delayed and performed after the patient has recovered from surgery, in which case it is referred to as secondary TEP. The choice of timing for performing the TEP and, consequently, the placement of the prosthesis is a matter of debate. It is estimated that primary TEP is performed in only 7.3% of patients undergoing total laryngectomy [16]. Despite studies associating primary TEP with the early recovery of the patient’s communicative capacity compared to secondary TEP [17], there is controversy regarding the indications, risks, and complications associated with each of these procedures [18]. This lack of consensus may be one of the reasons why secondary TEP continues to be the preferred option in the majority of cases (77.3%) when tracheoesophageal voice rehabilitation is proposed [19].

The development of these evidence-based recommendations aims to provide the necessary tools to professionals involved in patient selection and procedure execution to enhance the quality of life and care for patients undergoing total laryngectomy.

## 2. Methods

This manuscript is aimed at healthcare professionals involved in the diagnosis, treatment, rehabilitation, or follow-up of laryngectomized patients who are or could be users of voice prostheses: otorhinolaryngology specialists (physicians and residents), speech therapists, nursing staff, and other specialists. The objective is, through a systematic literature review, to define evidence-based recommendations regarding the procedure of primary TEP with voice prosthesis placement, establish the conditions and requirements for performing primary TEP, determine the indications and contraindications of primary TEP, as well as to define the complications and management of primary TEP.

The recommendations should be interpreted in the context of the individual needs and preferences of each patient within the specific healthcare setting of each case.

Given the continuous growth of knowledge and technological development, the recommendations will undergo periodic review from the moment of their publication.

The development process of this manuscript adhered to the Appraisal of Guidelines, Research and Evaluation II (AGREE II) guidelines [20]. To ensure the evidence-based support for the recommendations, a rapid systematic review was conducted following the recommended procedures (PROSPERO ID: CRD42024417529).

### 2.1. Search and Systematic Review

Evidence-based medicine methodologies were employed, following the PRISMA-RR, an extension to PRISMA (Preferred Reporting Items for Systematic Reviews and Meta-analysis) for rapid reviews and the following PICO framework [21,22,23]:

- Participants: Patients undergoing total laryngectomy.
- Intervention: Primary tracheoesophageal puncture.
- Comparison: Secondary tracheoesophageal puncture.
- Outcome/Results: Vocal outcomes, quality of life, and complications.

**Figure 1:**
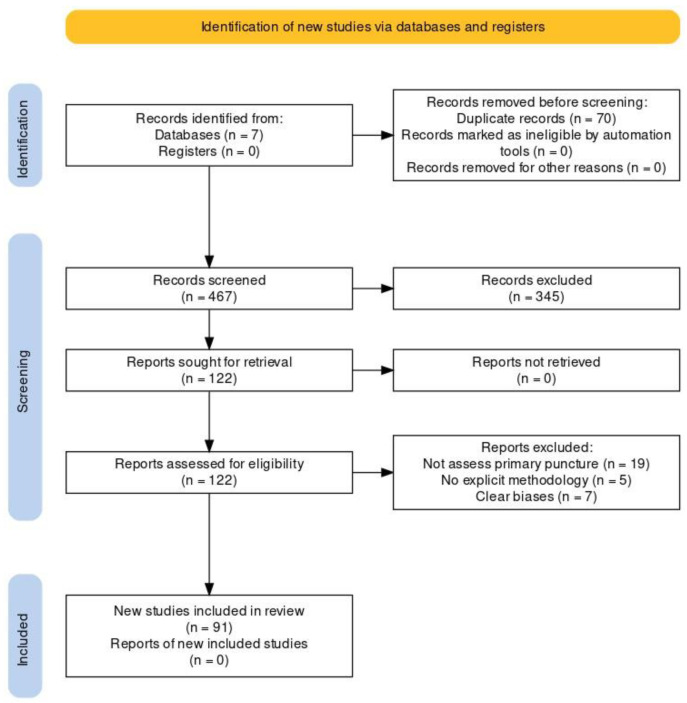
PRISMA flow diagram [24].

The search systematics were independently conducted by three of the authors (A.K.R; A.L.E; M.M.Y). Studies published in peer-reviewed scientific journals indexed in MEDLINE, Embase, Scopus, Web of Science, PubMed, Science Citation Index, and The Cochrane Library between 1 January 1980 and 1 January 2023 were included. The search terms used were voice prosthesis; total laryngectomy; tracheoesophageal puncture. The inclusion criteria considered randomized controlled trials, observational studies, meta-analyses, and systematic reviews published in English, Italian, Portuguese, and Spanish. Case reports, conference communications, or gray literature were not included (Figure 1). The recommendations and justifications were initially drafted and critically reviewed by the 3 previously mentioned authors. A mini-Delphi method with 2 meetings was used to define the recommendations. The recommendations were sent to the entire working group, who responded anonymously. In the first meeting, the recommendations with the proposed modifications were presented. In an open debate format, questions and other modifications were formulated. In the second meeting, the final points were reviewed and decided by consensus among the authors group. The group of authors consisted of 4 otolaryngologists and head and neck surgeons with clinical and scientific expertise in oncologic surgery, rehabilitation, and the use of voice prostheses. Additionally, 2 expert speech therapists in tracheoesophageal voice rehabilitation were part of the team.

### 2.2. Grading of Evidence and Recommendations

The Oxford Levels of Evidence system (2011, https://www.cebm.ox.ac.uk/resources/levels-of-evidence/ocebm-levels-of-evidence, accessed on 7 September 2023) was utilized for the grading of evidence and recommendations [25]. Recommendations were formulated based on the review and analysis of the most recent research on primary TEP found. During the formulation of recommendations for the management of primary TEP, all possible benefits, side effects, and risks were considered and clearly described with bibliographic references and supporting data. The nomenclature used to formulate the recommendations was (Level of Evidence, Grade of Recommendation). For example, (4, C).

### 2.3. Risk of Bias Analysis

Given the nature and characteristics of the studies obtained, the Risk Of Bias In Non-randomized Studies—of Exposure (ROBINS-E) tool was used (Supplementary Figure S1a,b). This tool provides a structured approach to assessing the risk of bias in observational epidemiological studies [26]. In cases where there was a control group compared to the intervention or in clinical trials, the ROBINS-I or RoB-2 tool was used depending on the type of study (Figure S2a,b) [27,28].

### 2.4. Recommendations Structure

In order to formulate recommendations that encompass the entire care process for primary TEP, the document was divided into the following sections:

- Primary Tracheoesophageal Puncture.
  ▪ Complementary Procedures to Facilitate Primary TEP and Voice Prosthesis Care.
- Primary TEP Indications.
- Primary TEP Contraindications.
- Benefits of Primary TEP over Secondary TEP.
  ▪ For the Patient.
  ▪ For the Professional.
  ▪ For the Healthcare System.
- Most Common Complications Related to Primary TEP.
  ▪ Leakage Around the Voice Prosthesis or Periprosthetic.
  ▪ TEP-Related Postoperative Infection.
  ▪ Stoma Stenosis.
- Influence of Primary TEP on the Occurrence of Postoperative Pharyngocutaneous Fistula.

## 3. Evidence-Based Recommendations

The different aspects found in the literature through the conducted systematic review are discussed. The formulated statements can be found collectively in Supplementary Table S1.

### 3.1. Primary Tracheoesophageal Puncture

Primary TEP is a surgical technique involving the creation of a tracheoesophageal fistula and the placement of a voice prosthesis (VP) during the same surgical procedure as total laryngectomy [29] (Supplementary Video S1). It is a safe and standardized procedure performed after the laryngeal resection is completed and before the closure of the pharyngeal mucosa (Video S1). Currently, for the safe, effective, and rapid execution of the procedure and voice prosthesis placement, it is recommended to use a kit marketed by the different manufacturing companies [30,31,32]. These insertion kits contain the sterile material necessary to perform the procedure. This approach aims to standardize the technique, make it reproducible, and minimize the risks of complications, regardless of the brand used (4, C) [33,34]. To date, there are no studies comparing one kit to another.

### Complementary Procedures to Facilitate the Primary TEP and VP Care

There are several surgical maneuvers that facilitate patient rehabilitation, reduce postoperative complications, and support postoperative VP and heat and moisture exchanger (HME) therapy management (Table 1) [35,36]. The most studied, and for which their implementation is recommended whenever possible, are detailed below.

The first one is related to the anchoring of the trachea and the formation of the stoma. Suturing the trachea laterally to the musculature prevents stoma stenosis or the stoma taking on an oval shape with a vertical longer axis closing the VP [37,38,39]. It is a simple technique, without described complications, and is reproducible. Other techniques have been described over the years, with none proving to be superior or undergoing comparative studies (4, C).

An excessively wide tracheostoma hinders manual closure for phonation or the use of HME adhesives [40]. It is recommended to adjust the size of the tracheostoma (1.5–2 cm) with a bevel tracheal incision. This provides a good exposure of the TEP, assists in the care of the VP, and facilitates its changes (4, C).

The cricopharyngeal muscle is located superiorly to the tracheoesophageal fistula and can impede phonation and swallowing if it undergoes hyperfunction [41]. Performed in nearly half of patients [16], a cricopharyngeal myotomy reduces pressure on the upper esophageal sphincter during swallowing or phonation [42]. It is considered a recommended and safe surgical maneuver for experienced professionals, although not without complications [43,44,45] (4, C).

Finally, the section of the medial portion of both sternocleidomastoid muscles at their distal end promotes flattening of the tracheostoma, facilitates VP management, and enhances adherence to adhesives and HME. It is a simple, quick, and safe procedure [46,47,48] (4, C).

### 3.2. Primary TEP Indications

There is no scientific evidence to establish absolute indications for the performance of primary TEP at present (Table 2) [7,49]. Given the potential benefits of primary TEP, the possibility of performing primary TEP should be considered in every patient undergoing total laryngectomy [50]. A personalized assessment of each surgical situation is necessary when deciding the timing of TEP [50,51,52,53], with a positive preoperative multidisciplinary and multidimensional assessment (speech therapy, psychology, otorhinolaryngology, social worker’s assistant) [16,54,55,56] (3b, B). The relative indications for primary TEP could be divided according to local or general factors.

Considering local factors, primary TEP can be performed in patients with either tumor-related or non-tumor-related pathologies requiring total laryngectomy [57] (4, C). Additionally, primary TEP can be performed in tumors with extensive resections and reconstructions of the pharyngoesophageal segment, using both pedicled and free flaps [16,51,58,59,60,61,62] (4, C). In cases of surgical salvage after chemoradiotherapy (CRT), primary TEP has not been shown to significantly increase complications (4, C). Nevertheless, some studies suggest a higher incidence of complications with salvage surgery and not with TEP and VP placement, especially after radiotherapy [51,52,54,60,63].

Regarding general factors, a good overall patient condition without incapacitating comorbidities is required [64,65]. Specifically, there should be no advanced lung pathology, as minimal lung volumes and capacities are necessary to expel the airflow required for voice prosthesis phonation [58,60] (4, C). The necessary physical capacity and manual coordination to effectively occlude the stoma [54,55,58] are also crucial (4, C). Additionally, a minimum level of visual capacity is needed for prosthesis care if there is no adequate social or family support [54,58] (4, C). Patient motivation for prosthesis management and self-care is fundamental for rehabilitative success [55,56,59,64,66,67,68] (3a, B), and having good social/family support is helpful [16,54,69] (3a, B).

Finally, the experience of the hospital center in the technique and having the necessary resources for follow-up, both from the healthcare system and the patient, is key to rehabilitative success [16,54,64,65,70] (2b, C).

### 3.3. Primary TEP Contraindications

There are no absolute contraindications for primary TEP at present [7,49]. Relative contraindications for primary TEP can also be divided based on local or general factors and are complementary to the indications previously mentioned (Table 3).

Among local factors, extensive lingual or mandibular involvement requiring large resections leads to ankyloglossia or trismus after treatment. This inability to articulate words may be considered a contraindication for primary tracheoesophageal puncture. [54] (4, C). Previous treatment with CRT, as mentioned earlier, does not significantly increase the incidence of complications associated with primary TEP [51,71,72]. Nevertheless, in selected cases with a high risk of postoperative complications, where the neck has previously received radiation therapy (presence of radionecrosis or generalized fibrosis), or where severe postoperative medical–surgical complications are anticipated, it may be preferable to perform secondary TEP [52,54,60,73] (4, C).

Among the general factors to consider with potential complications is the overall poor health of the patient, with incapacitating comorbidities preventing proper rehabilitation. The presence of distant metastasis without the possibility of curative treatment or a reduced life expectancy is also a factor to consider in the choice of primary TEP. Advanced lung disease, physical incapacity (poor manual and visual coordination), lack of motivation on the part of the patient [74], lack of social/family support [56,69,75], lack of resources/experience in the hospital center, or difficult access to healthcare for the patient are other factors that could influence the decision to undergo TEP (4, C).

### 3.4. Benefits of Primary TEP over Secondary TEP

Primary TEP involves a single surgery that avoids the need for a new admission and postoperative complications associated with secondary TEP [16,17,52,62,74] (4, C). The studies indicate that the placement of the VP during surgery is related to significantly fewer early device changes (1.4 vs. 2), fewer VP changes due to resizing (8% vs. 80%), longer durations before the initial VP change (159.7 vs. 24.5 days), an earlier start to voice rehabilitation (13.2 vs. 17.6 days), a shorter hospital stay (17.2 vs. 24.5 days), and cost savings of USD 559.83 per person.

Therefore, the performance of primary TEP entails a series of benefits that can be divided into three groups: for the patient, for the professional, and for the healthcare system.

#### 3.4.1. For the Patient

Primary TEP does not appear to entail greater surgical complications or higher morbidity than secondary TEP [51,76,77] (4, C). These statements are based on observational studies and should be taken with caution.

Primary TEP seems to improve Voice-Related Quality of Life [6,12,13,69] (4, C) thanks to the following:

- Earlier communication with primary TEP compared to secondary TEP [17,49,52,54,55,59,71,78] (4, C).
- The early initiation of rehabilitation and achieving proper phonation before receiving supplementary RT treatment if necessary [79,80,81] (4, C). Rehabilitation can begin around two weeks (10–14th day) after surgery if there are no complications associated with the procedure [52,82,83] (4, C). The time to achieve fluent phonation after a total laryngectomy is around 56 days in the case of primary TEP and 200 days in the case of secondary TEP, respectively [61].
- Quicker familiarization with the prosthesis, phonation, and care compared to secondary TEP [73].
- The results on the Harrison–Robillard-Schultz Tracheoesophageal Rating Scale do not differ between primary and secondary TEP, with scores of >11 in both cases [84,85] (4, C).
- Primary TEP is associated with an earlier return to work for active workers [86] (4, C).

Regarding postoperative recovery, the primary TEP technique is associated with greater plasticity of the pharyngoesophageal sphincter, facilitating better short- and long-term voice outcomes [52,59,74] (4, C). Likewise, the primary TEP technique supposes better stabilization and earlier healing of the tracheoesophageal fistula [16,17,62,87] (4, C).

#### 3.4.2. For the Professional

Primary TEP is associated with less frequent prosthesis replacements, the longer duration of the first prosthesis compared to a VP of secondary TEP [17,33,51] (4, C), a reduced frequency of follow-up consultations [33,59] (4, C), and a lower risk of esophageal perforation, avoiding rigid esophagoscopy in laryngectomized patients who may have esophageal stenosis. 

Pharyngeal stenosis associated with RT can complicate the secondary puncture technique and phonatory rehabilitation [16,59] (4, C). The primary TEP technique supposes better accessibility and surgical ease in creating the fistula in a more natural and horizontal position, which can subsequently facilitate VP replacements [32,33] (4, C). As mentioned previously, the primary TEP technique adds the possibility of performing complementary surgical techniques to facilitate phonation or VP replacement [16,17,33,62] (4, C).

#### 3.4.3. For the Healthcare System (4, C)

The performance of a single procedure implies the avoidance of a new admission, intervention, and postoperative complications [16,33]. The primary TEP seems to reduce the need for prosthesis replacements and lower the requirement for follow-up consultations and rehabilitation sessions [17], with consequent cost savings for the system [51].

### 3.5. Most Common Complications Related to Primary TEP

The most common complications associated with primary TEP appear to be leakage around the prosthesis, postoperative infection, and stoma stenosis, with a wide variability among the analyzed series [52,73,88] (Table 4). In a systematic review and meta-analysis, it was observed that there were no statistically significant differences in the percentage of postoperative infection (primary TEP: 9.1% vs. secondary TEP: 3.9%) or stoma stenosis (primary TEP: 8.5% vs. secondary TEP: 4.5%) between patients in both groups. However, statistically significant differences were found in the percentage of leakage around the prosthesis, with a 10% reduction in the case of secondary puncture (primary TEP: 22.5% vs. secondary TEP: 6.5%) [16,88]. Given the heterogeneity of the data, a causal relationship between primary TEP and an increase in the presentation of this type of complication cannot currently be established [52,73,88].

#### 3.5.1. Leakage around the VP or Periprosthetic

Leakage around the VP or periprosthetic is the most frequent complication after performing a primary TEP [87]. Due to this, there is an aspiration of saliva, liquids, or food around the prosthesis, which increases the risk of pneumonia and respiratory complications [89,90]. Systematic reviews and meta-analyses suggest that the appearance of this type of complication is related to an advanced nodal stage, postoperative pharyngeal stenosis, the presence of pharyngoesophageal reflux, postoperative radiation therapy, or locoregional recurrence or metastatic cancer after laryngectomy [67,87,90].

The management of this complication is usually conservative [35,91,92,93] (4, C). Initially, techniques such as adjusting the size of the prosthesis or placing a silicone ring around the tracheal face of the prosthesis are used [94,95]. Replacement with a double-flanged prosthesis (Provox^®^ Vega™ XtraSeal™) has shown a reduction in periprosthetic leakage (9.62% with XtraSeal vs. 22.43% in the control group) and has been shown to be a cost-effective procedure in the long term [63,96,97] (3a, B). The Blom-Singer large oesophageal and tracheal flange VP is also a useful solution for the management of periprosthetic leakage, ensuring similar voice quality and an identical lifespan to that of other voice prostheses [95] (4, C).

Other frequently used techniques include temporary removal of the VP, circular suture of the fistula, or tissue augmentation with the injection around the fistula of different materials (collagen, bioplastics, autologous fat, hyaluronic acid…). These methods aim to reduce the diameter of the fistula [87,98] (4, C). Closing the tracheoesophageal fistula by placing a silicone prosthesis or surgically are alternatives when conservative options have failed [99]. These techniques achieve control of leakage but impair phonation, with a negative impact on patients’ quality of life [100,101] (4, C). 

#### 3.5.2. TEP-Related Postoperative Infection (4, C)

Peristomal infection is the most frequent infection, followed by cervical cellulitis. There are cases of deep cervical infections, abscesses, infections in the cervical spine, or complications such as mediastinitis, but there is no evidence that they are associated with primary TEP to a greater extent than with secondary. There appears to be a greater tendency for local infection in patients undergoing primary TEP compared to those who do not undergo any procedure [52,102,103]. The management of these complications is usually conservative, with appropriate antibiotic therapy and admission if necessary [89,100]. Primary TEP does not negatively affect the incidence of postoperative medical complications and the duration of hospitalization [102].

#### 3.5.3. Stoma Stenosis (4, C)

There is weak evidence suggesting that primary TEP may be associated with stoma stenosis [103]. This could be related to an increased risk of postoperative local infection. A stenosed tracheostoma can complicate the maintenance of the voice prosthesis and potential replacements. To prevent this, some recommendations during the laryngotracheoplasty have been presented earlier in this document. In the case of stoma stenosis, the treatment is surgical and involves performing a stomaplasty [104].

### 3.6. Influence of Primary TEP on the Occurrence of Postoperative Pharyngocutaneous Fistula

There is some controversy regarding the influence of primary TEP on the occurrence of postoperative pharyngocutaneous fistula after total laryngectomy [73,103] (Table 5). In the majority of studies conducted, statistically significant differences were not found between the increased incidence of pharyngocutaneous fistula and primary TEP [19,105,106,107,108,109], nor in more recent meta-analyses [52,88] (4, C).

## 4. Limitations

It is important to acknowledge the inherent limitations of these recommendations, as the majority of the included studies exhibit a limited level of quality. The predominant evidence is characterized by a low level (4), which may impact the robustness of the formulated recommendations. Despite the abundance of articles on voice prostheses, few specifically evaluate primary puncture versus secondary puncture. Most of them are observational, retrospective, and have a limited sample size. 

Similarly, an important aspect lacking in studies on tracheoesophageal VP is the uniformity of care for patients with TEP. This aspect has been highlighted by various working groups previously [83,91,92,110]. There are multiple factors that can affect TEPs which are not considered in the selected studies, such as the location of the institution where the study is conducted, economic level, access to healthcare, cultural level, etc. [111].

Given the lack of high-quality studies, the interpretation of results and the generalization of conclusions may be subject to a higher degree of uncertainty. The need for future research with more rigorous designs and robust methodologies is evident to strengthen the evidence base and enhance the validity of the recommendations provided.

## 5. Conclusions

This manuscript defines the surgical protocol for primary TEP and voice prosthesis placement. It also establishes recommendations for primary TEP (indications, contraindications, initiation of phonation, and complications) based on current scientific evidence. The recommendations should be interpreted in the context of the individual needs and preferences of each patient within the specific healthcare setting of each case.

A total of 19 statements were formulated, with 78.95% of them having a Level of Evidence of 4 and a Grade of Recommendation of C. Only four statements reached a Grade of Recommendation of B, while two had a 2b evidence level and 2 had a 3b evidence level.

There is not sufficient evidence comparing the outcomes of primary TEP versus secondary TEP. Future studies with robust methodologies are needed to clarify the role of primary and secondary TEP in the rehabilitation of patients undergoing total laryngectomy.

## Figures and Tables

**Table 1:** Summary of statements related to the techniques for performing primary TEP.

Number	Statement	Level of Evidence	Grade of Recommendation
1	The use of commercially available kits for the performance of primary TEP is recommended.	4	C
2	Tracheal suturing to the lateral musculature is recommended to prevent stoma stenosis.	4	C
3	A stoma size between 1.5 and 2 cm is recommended for improved use, care, and replacement of the VP, as well as HME therapy.	4	C
4	Cricopharyngeal myotomy is recommended to reduce swallowing and phonation pressure.	4	C
5	The section of the medial portion of both sternocleidomastoid muscles at their distal end is recommended to flatten the stoma.	4	C

Abbreviations: TEP, tracheoesophageal puncture; HME, heat and moisture exchanger.

**Table 2:** Summary of statements related to primary TEP indications.

Number	Statement	Level of Evidence	Grade of Recommendation
6	Primary TEP can be performed in all laryngectomized patients, regardless of the location and extent of the tumor or the need for reconstructions with free or pedicled flaps.	4	C
7	Primary TEP in salvage total laryngectomies after chemoradiotherapy has not been shown to increase the incidence of complications related to the VP.	4	C
8	A multidisciplinary and multidimensional preoperative evaluation is recommended to correctly select candidates for primary TEP. This evaluation should include an assessment of the patient’s overall health, motivation, speech therapy evaluation, ORL evaluation, and evaluation of social/family support.	3b	B
9	The best rehabilitative outcomes are found in centers with experience, high specialization, and sufficient resources and patient volume.	2b	B

Abbreviations: TEP, tracheoesophageal puncture; VP, voice prosthesis; ORL, otorhinolaryngology.

**Table 3:** Summary of statements related to primary TEP contraindications.

Number	Statement	Level of Evidence	Grade of Recommendation
10	Primary TEP is not recommended in cases of lingual or mandibular involvement requiring total glossectomy or resulting in sequelae that prevent proper word articulation.	4	C
11	Primary TEP is not recommended for patients in poor overall health, with incapacitating comorbidities, a lack of motivation for rehabilitation, or a negative assessment following preoperative evaluation.	4	C
12	In a patient at high risk of postoperative complications, including pharyngocutaneous fistula, deferring the performance of primary TEP should be considered.	4	C
13	The performance of primary TEP or rehabilitation with tracheoesophageal voice in healthcare centers without the necessary resources for proper rehabilitative treatment and follow-up is not recommended.	2b	B

Abbreviations: TEP, tracheoesophageal puncture.

**Table 4:** Summary of statements related to primary TEP complications.

Number	Statement	Level of Evidence	Grade of Recommendation
14	The treatment of periprosthetic leakage should be gradual and systematic, escalating interventions from more conservative to more aggressive.	4	C
15	For the management of periprosthetic leakage, the replacement of the prosthesis with a double-flanged one, such as the Provox^®^ Vega™ XtraSeal™, is recommended.	3b	B
16	For the management of periprosthetic leakage, VP replacement with the adjustment of diameter and length, or the placement of a silicone sheet on the tracheal side of the prosthesis, is also recommended.	4	C
17	For the treatment of local infection in postoperative tracheoesophageal fistula, initiating a conservative approach with antibiotics and ongoing monitoring is recommended.	4	C
18	Surgical stomaplasty is recommended for the treatment of respiratory stoma stenosis.	4	C

Abbreviations: VP, voice prosthesis.

**Table 5:** Statement of the relation of primary TEP and postoperative pharyngocutaneous fistula.

Number	Statement	Level of Evidence	Grade of Recommendation
19	The performance of primary TEP has not been shown to influence the incidence of pharyngocutaneous fistula following total laryngectomy.	4	C

Abbreviations: TEP, tracheoesophageal puncture.

## Data Availability

Data are contained within the article or Supplementary Materials.

## Supplementary Materials

The following supporting information can be downloaded at: https://www.mdpi.com/article/10.3390/healthcare12060652/s1, Video S1: Primary TEP procedure; Figure S1: Risk of bias assessment ROBINS-E tool; Figure S2: Risk of bias assessment ROBINS-I/Rob-2 tool. Table S1: Summary of statements.
