# Supplementary material for: Evidence-Based Recommendations in Primary Tracheoesophageal Puncture for Voice Prosthesis Rehabilitation
Source: Healthcare (Basel). 2024 Mar 14;12(6):652. doi: 10.3390/healthcare12060652 (PMC10970215; doi:10.3390/healthcare12060652)
Supplement: Supplementary file 1 [file healthcare-12-00652-s001.zip › Table S1.pdf]

Supplementary Table S1. Summary of Statements.

Number	Statement	Level of Evidence	Grade of Recommendation
1	The use of commercially available kits for the performance of primary TEP is recommended.	4	C
2	Tracheal suturing to the lateral musculature is recommended to prevent stoma stenosis.	4	C
3	A stoma size between 1.5 - 2 cm is recommended for improved use, care, and replacement of the VP, as well as HME therapy.	4	C
4	Cricopharyngeal myotomy is recommended to reduce swallowing and phonation pressure.	4	C
5	The section of the medial portion of both sternocleidomastoid muscles at their distal end is recommended to flatten the stoma.	4	C
6	Primary TEP can be performed in all laryngectomized patients, regardless of the location and extent of the tumor or the need for reconstructions with free or pedicled flaps.	4	C
7	Primary TEP in salvage total laryngectomies after chemoradiotherapy has not been shown to increase the incidence of complications related to the VP.	4	C
8	A multidisciplinary and multidimensional preoperative evaluation is recommended to correctly select candidates for primary TEP. This evaluation should include an assessment of the patient's overall health, motivation, speech therapy evaluation, ORL evaluation, and evaluation of social/family support.	3b	B
9	The best rehabilitative outcomes are found in centers with experience, high specialization, and sufficient resources and patient volume.	2b	B
10	Primary TEP is not recommended in cases of lingual or mandibular involvement requiring total glossectomy or resulting in sequelae that prevent proper word articulation.	4	C
11	Primary TEP is not recommended for patients in poor overall health, with incapacitating comorbidities, a lack of motivation for rehabilitation, or a negative assessment following preoperative evaluation.	4	C
12	In a patient at high risk of postoperative complications, including pharyngocutaneous fistula, deferring the performance of primary TEP should be considered.	4	C
13	The performance of primary TEP or rehabilitation with tracheoesophageal voice in healthcare centers without the necessary resources for proper rehabilitative treatment and follow-up is not recommended.	2b	B
14	The treatment of periprosthetic leakage should be gradual and systematic, escalating interventions from more conservative to more aggressive.	4	C
15	For the management of periprosthetic leakage, the replacement of the prosthesis with a double-flanged one,	3b	B

	such as the Provox® Vega™ XtraSeal™, is recommended.		
16	For the management of periprosthetic leakage, VP replacement with adjustment of diameter and length, or the placement of a silicone sheet on the tracheal side of the prosthesis, is also recommended.	4	C
17	For the treatment of local infection in postoperative tracheoesophageal fistula, initiating a conservative approach with antibiotics and ongoing monitoring is recommended.	4	C
18	Surgical stoma-plasty is recommended for the treatment of respiratory stoma stenosis.	4	C
19	The performance of primary TEP has not been shown to influence the incidence of pharyngocutaneous fistula following total laryngectomy	4	C

Abbreviations: TEP, tracheoesophageal puncture; HME, Heat and Moisture Exchangers; VP, Voice Prosthesis; ORL, Otorhinolaryngology
