# Supplementary figures and images for: Evidence-Based Recommendations in Primary Tracheoesophageal Puncture for Voice Prosthesis Rehabilitation
Source: Healthcare (Basel). 2024 Mar 14;12(6):652. doi: 10.3390/healthcare12060652 (PMC10970215; doi:10.3390/healthcare12060652)

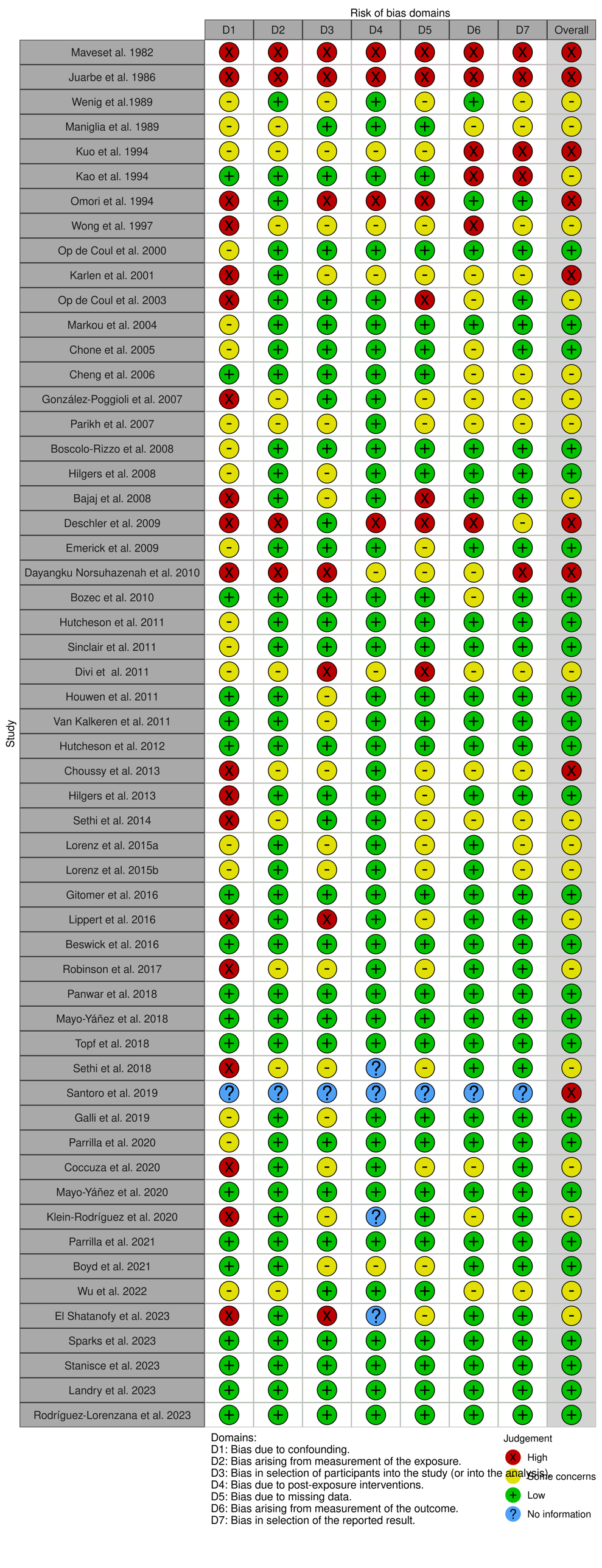

Supplement: Supplementary file 1 [file healthcare-12-00652-s001.zip › Figure S1a.png]

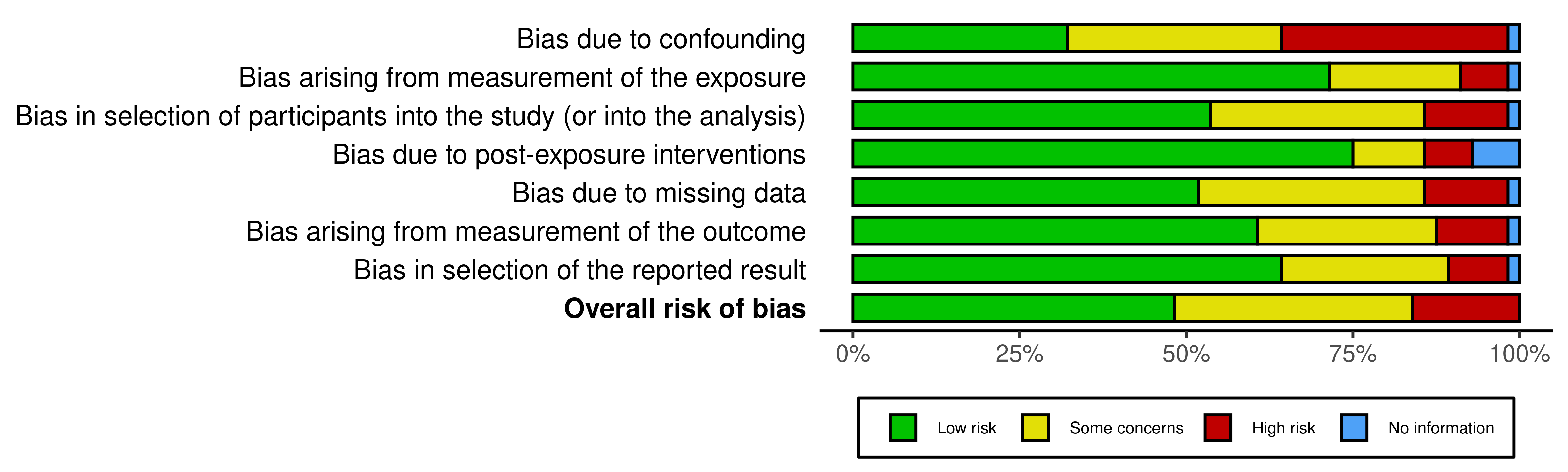

Supplement: Supplementary file 1 [file healthcare-12-00652-s001.zip › Figure S1b.png]

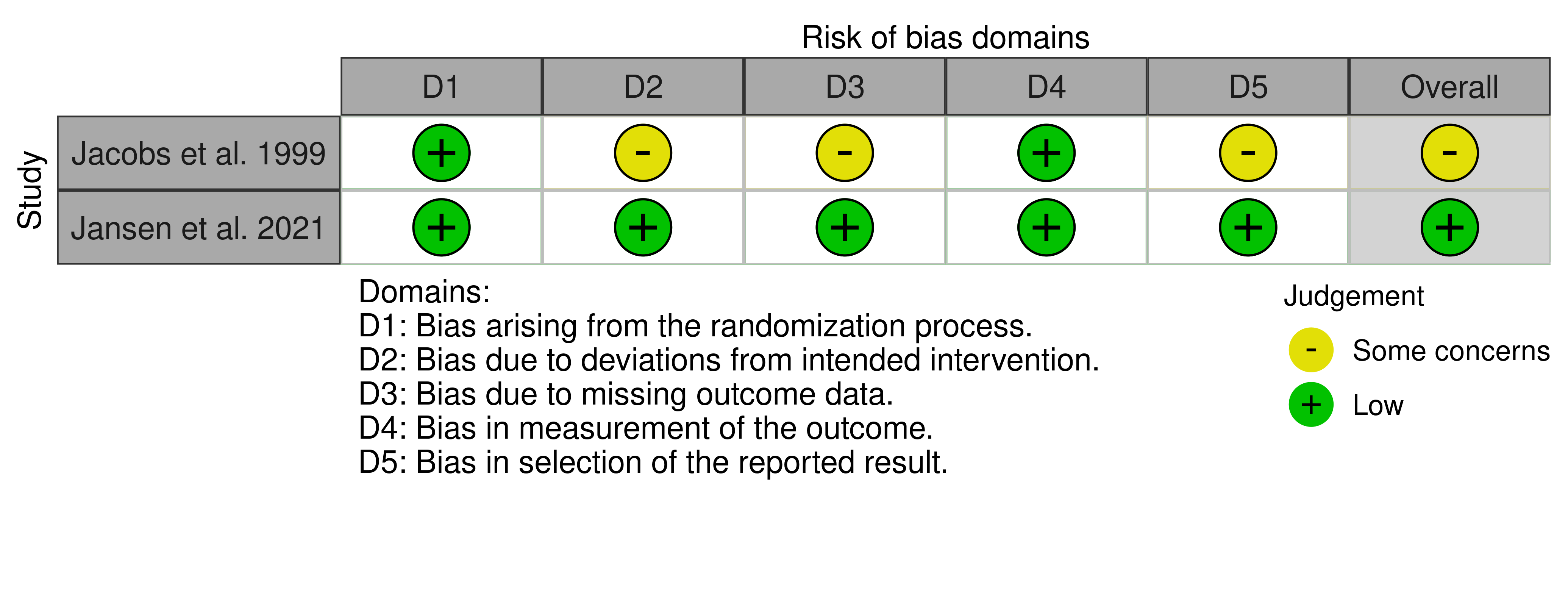

Supplement: Supplementary file 1 [file healthcare-12-00652-s001.zip › Figure S2a.png]

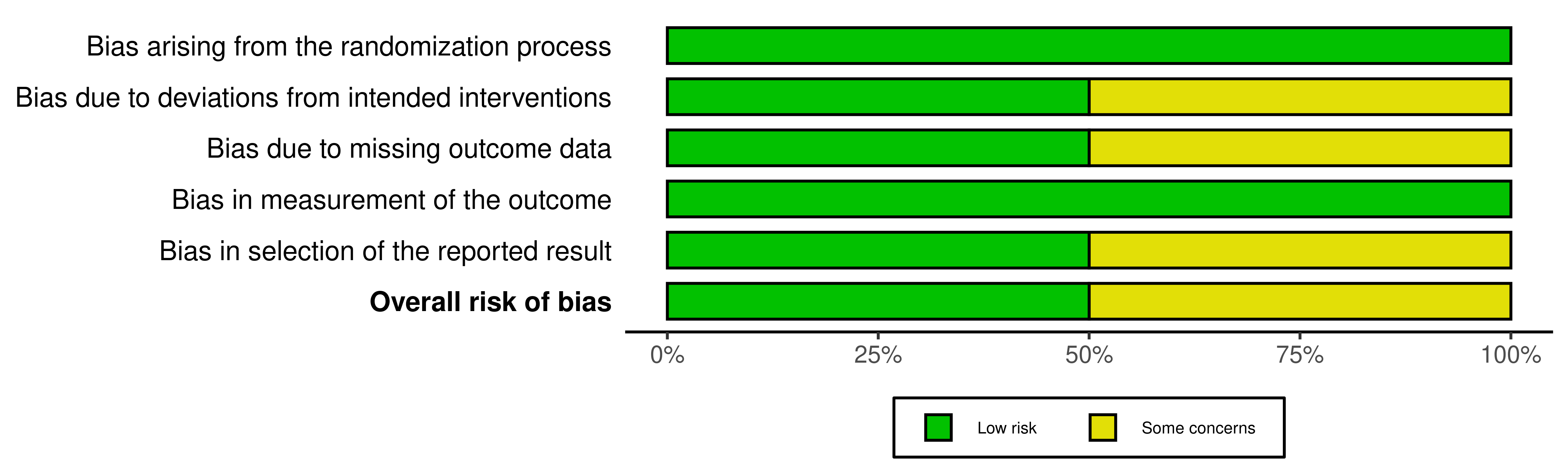

Supplement: Supplementary file 1 [file healthcare-12-00652-s001.zip › Figure S2b.png]
